# Supplementary material for: One-volt-driven superfast polymer actuators based on single-ion conductors
Source: Nat Commun. 2016 Nov 18;7:13576. doi: 10.1038/ncomms13576 (PMC5120218; doi:10.1038/ncomms13576)
Supplement: Supplementary Information — Supplementary Figure 1-6 [file ncomms13576-s1.pdf]

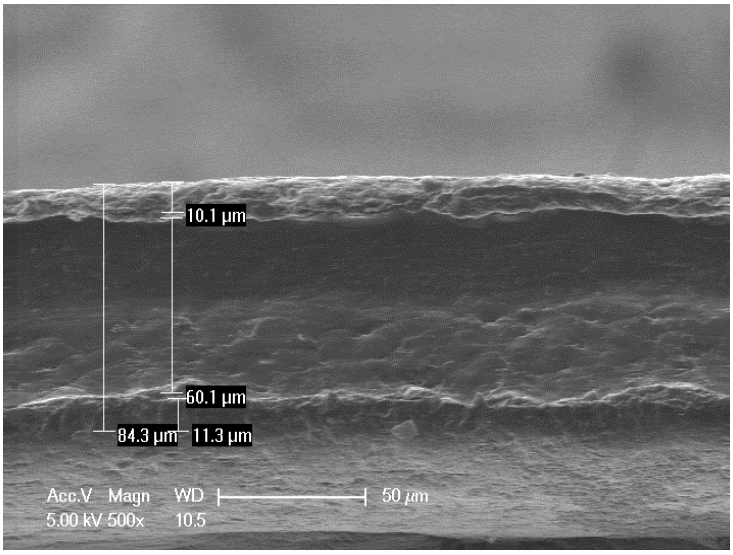

Supplementary Figure 1: Cross-sectional SEM image of the actuator in a tri-laminar structure. For the fabrication of actuators, approximately 60 μm thick cation-conducting polymer membranes were sandwiched between 10 μm thick single-walled carbon nanotubes (SWCNT) electrodes via hot pressing.

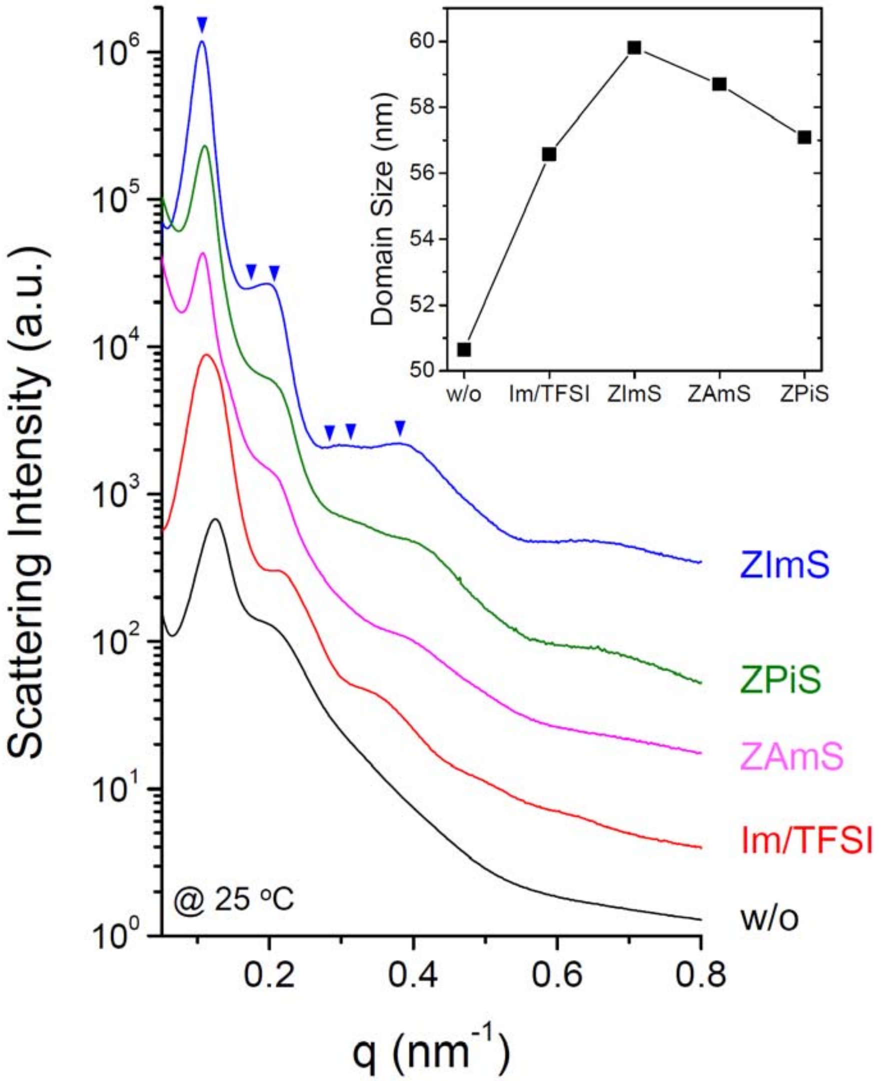

Supplementary Figure 2: Morphology of Im-doped $S_{153}MB_{313}$(60) in the absence and presence of ionic additives. SAXS profiles of Im-doped $S_{153}MB_{313}$(60) with the addition of zwitterions or ionic liquid, measured at room temperature. The type of introduced ionic additives is noted in the figure. Representative Bragg peaks at $q^*, \sqrt{3}\,q^*, \sqrt{4}\,q^*, \sqrt{7}\,q^*, \sqrt{9}\,q^*, \sqrt{12}q^*$ (▼) for Im-doped $S_{153}MB_{313}$(60) containing ZImS, indicative of HEX structure, are marked in the figure. The changes in domain size with ionic additives are also plotted in the inset.

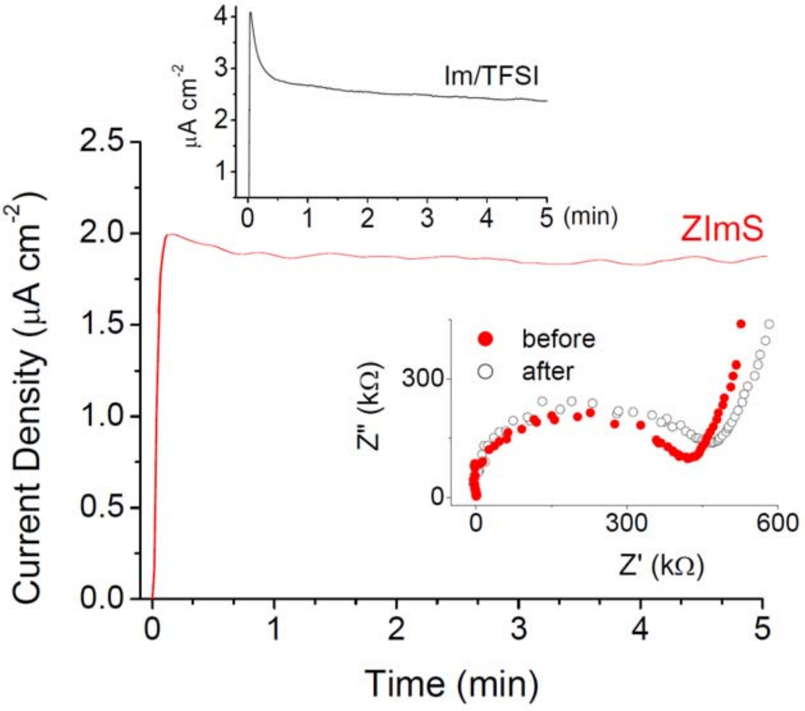

Supplementary Figure 3: Single-ion conducting properties of $S_{153}MB_{313}(60)$ containing ZImS. Current density profiles of Im-doped $S_{153}MB_{313}(60)$ containing ZImS, compared to Im/TFSI-embedded analog, after polarizing the samples with ΔV of 50 mV. The ratio of the current flow at steady state to the initial current flow (Iss/I_0) was determined to be 0.88 for Im-doped $S_{153}MB_{313}(60)$ containing ZImS, which far exceeds the low value of 0.58 of Im/TFSI-embedded analog. The non-unity Iss/I_0 of the sample containing ZImS is related to the relaxation of the sulfonate anion tethered in the polymer backbone. Nyquist plots of the sample containing ZImS obtained before and after the polarization are provided in the inset.

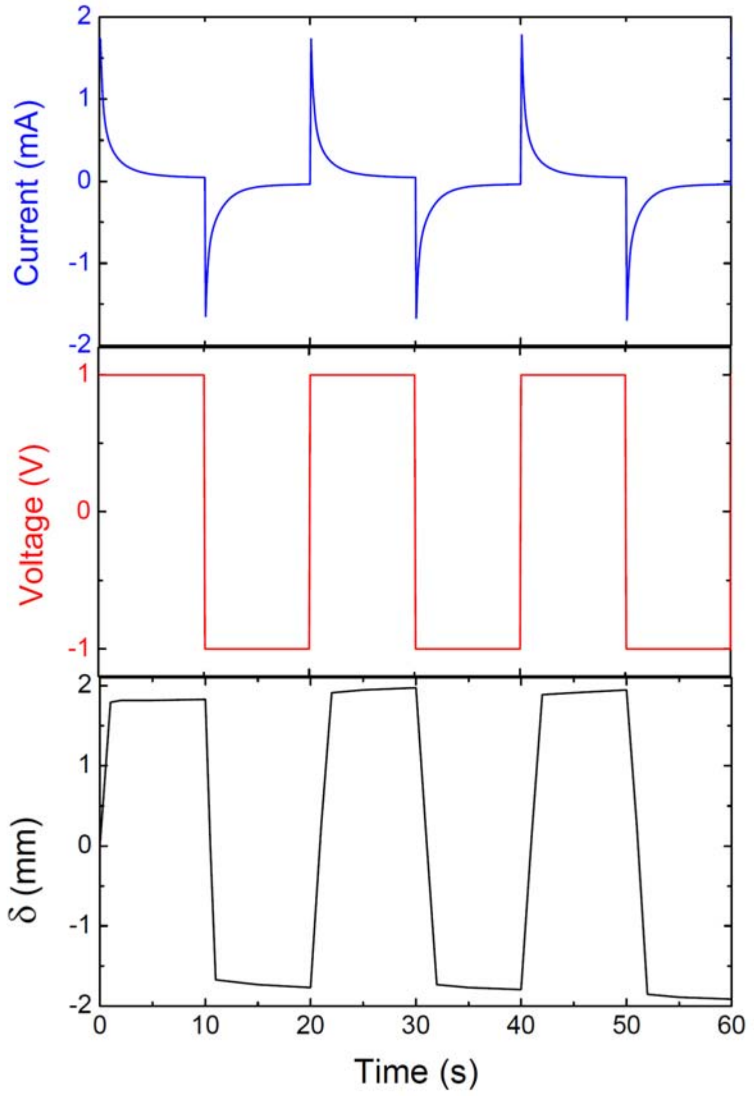

Supplementary Figure 4: Current, voltage, and displacement of the actuator. The current-voltage-displacement response of the actuator based on Im-doped $S_{153}MB_{313}(60)$ containing ZImS at alternating square-wave voltages of ± 1 V and a frequency of 0.05 Hz.

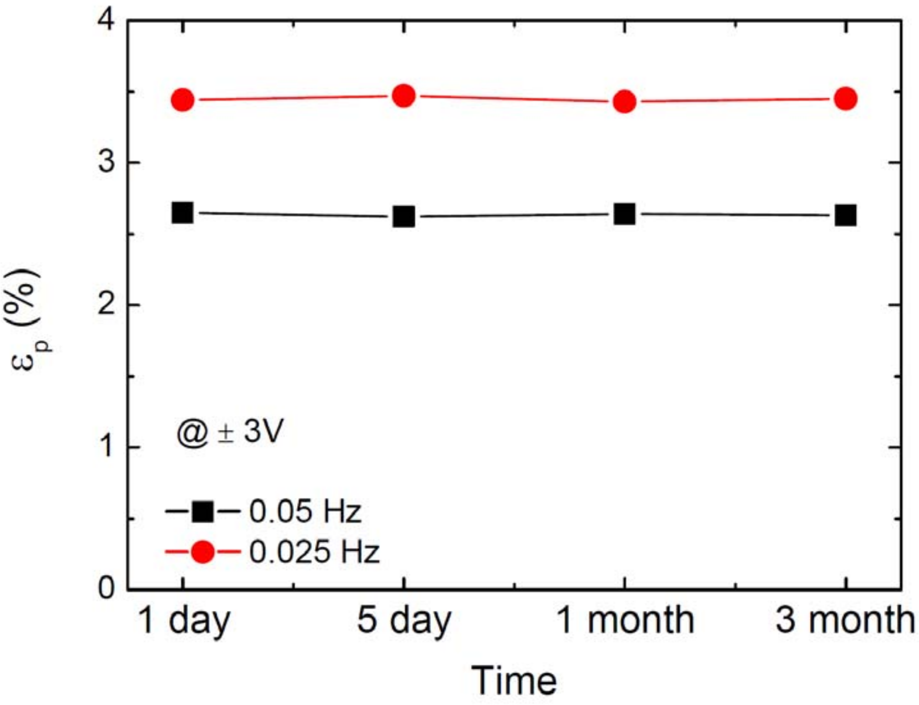

Supplementary Figure 5: Time-dependent bending strain of the actuator. The peak-to-peak bending strain (ε_p) of the actuator containing ZImS with extended time periods, measured at alternating square-wave voltages of ± 3 V and frequencies of 0.05 Hz and 0.025 Hz, confirming the negligible leaching of ionic additives from the actuator.

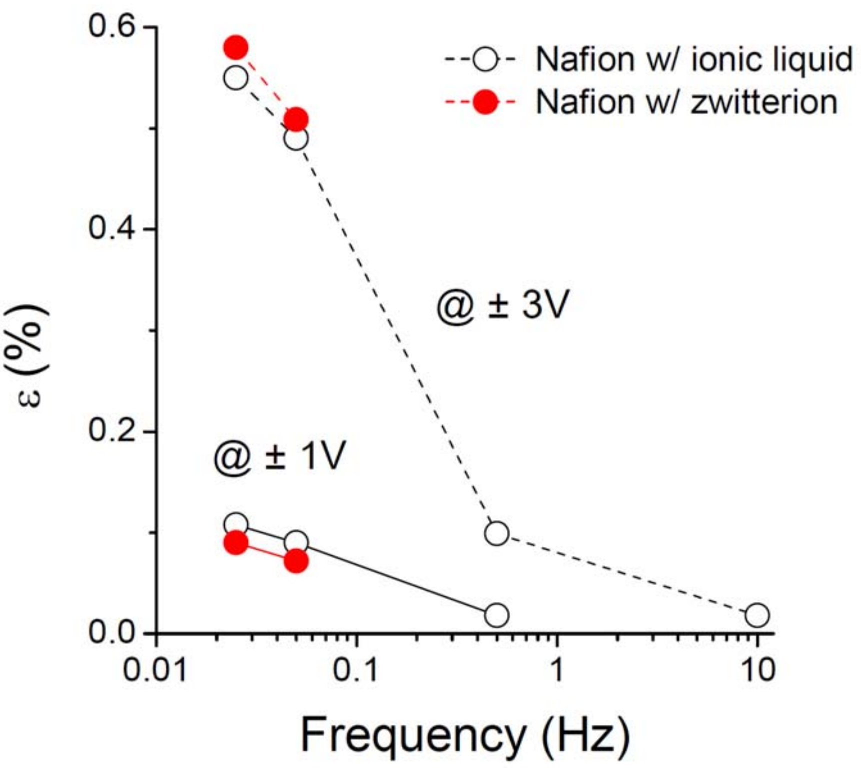

Supplementary Figure 6: Performance of Nafion^{TM} actuators containing ionic additives. The bending strain (ε) of the Nafion^{TM} actuators at alternating square-wave voltages of ± 3 V and ± 1 V, indicating large reductions in the ε values along with slower response time upon decreasing operation voltage.
